# P02.153. Effectiveness of biofield therapy for patients with sickle cell disease in Africa

**DOI:** 10.1186/1472-6882-12-S1-P209

**Published:** 2012-06-12

**Authors:** M Mikobi, K Suzuki

**Affiliations:** 1 The Medical Center for Sickle Cell Anemia, Kinshasa, Congo; 2 MOA Health Science Foundation, Tokyo, Japan

## Purpose

Patients with sickle cell disease (SCD) in Africa have a high risk of premature death, mainly due to insufficient medical services. Some SCD patients in the first author’s clinic experienced symptomatic improvement after administration of biofield therapy. The objective of this study was to evaluate the effectiveness of biofield therapy for SCD patients over a 4-year period.

## Methods

We adopted Okada Purifying Therapy (OPT) as an intervention in this study. We formed a group of 20 SCD patients aged 3-36 years (OPT group), and a control group (n=20) of a matching age/gender profile from regular patients at the clinic. OPT was administered by certified practitioners approved by MOA International Corporation (http://www.moainternational.or.jp/) every weekday for one year. We examined participating patients’ blood tests at the beginning and end of the 1-year period and ran a follow-up survey after 4 years.

## Results

During the 1-year study period, the OPT group showed a significant improvement in the blood data: hemoglobin [median value from 6.3g/dl (25-75 percentile: 6.1-7.2) to 10.1g/dl (9.4-11.1), p<0.001], total bilirubin [from 9.3mg/dl (6.5-9.4) to 3.2mg/dl (2.2-4.1), p<0.001], and creatinine [from 1.1mg/dl (0.9-1.7) to 0.7mg/dl (0.6-0.8), p<0.001]. The control group had less improvement in hemoglobin [from 6.3g/dl (6.0-7.0) to 7.1g/dl (6.4-7.9), p=0.015] and total bilirubin [from 9.1mg/dl (6.8-9.3) to 6.8mg/dl (6.2-7.6), p=0.004] than the OPT group (p<0.001); creatinine increased from 0.9mg/dl (0.8-1.1) to 1.2mg/dl (1.0-1.5) (p<0.001). Three patients in the OPT group and all of the control group needed hospitalization once or more (p<0.001). After 4 years, 17 in the OPT group and 13 in the control group survived (p=0.27).

## Conclusion

Considering the insufficient medical services, biofield therapy has possibly contributed to the positive outcomes, although the care given by the practitioners may also have had some influences. In conclusion, biofield therapy is considered safe and effective for SCD patients with various symptoms.

